# C-phycocyanin: a natural product with radiosensitizing property for enhancement of colon cancer radiation therapy efficacy through inhibition of COX-2 expression

**DOI:** 10.1038/s41598-019-55605-w

**Published:** 2019-12-16

**Authors:** Amirhosein Kefayat, Fatemeh Ghahremani, Ashkan Safavi, Alireza Hajiaghababa, Jamal Moshtaghian

**Affiliations:** 1 Department of Oncology, Cancer Prevention Research Center, Isfahan University of Medical Sciences, Isfahan, 81746-73461 Iran; 2 Department of Medical Physics and Radiotherapy, Arak University of Medical Sciences, Arak, 38481-76941 Iran; 3 Department of Biology, Science and Research Branch, Islamic Azad University, Tehran, Iran; 4 Department of Biology, Payame Noor Isfahan University, Isfahan, Iran; 5 Division of Cell and Molecular Biology, Department of Biology, Faculty of Science, University of Isfahan, Isfahan, Iran

**Keywords:** Colon cancer, Radiotherapy

## Abstract

Different chemical and nanomaterial agents have been introduced for radiosensitizing purposes. However, many researchers believe these agents are far away from clinical application due to side effects and limited knowledge about their behavior in the human body. In this study, C-phycocyanin (C-PC) was used as a natural radiosensitizer for enhancement of radiation therapy (RT) efficacy. C-PC treatment’s effect on the COX-2 expression of cancer cells was investigated by flow cytometry, western blot, qRT-PCR analyses *in vitro* and *in vivo*. Subsequently, the radiosensitizing effect of C-PC treatment was investigated by MTT and clonogenic cell survival assays for CT-26, DLD-1, HT-29 colon cancer cell lines and the CRL-1831 as normal colonic cells. In addition, the C-PC treatment effect on the radiation therapy efficacy was evaluated according to CT-26 tumor’s growth progression and immunohistochemistry analyses of Ki-67 labeling index. C-PC treatment (200 µg/mL) could significantly enhance the radiation therapy efficacy *in vitro* and *in vivo*. Synergistic interaction was detected at C-PC and radiation beams co-treatment based on Chou and Talalay formula (combination index <1), especially at 200 µg/mL C-PC and 6 Gy radiation dosages. The acquired DEF of C-PC treatment was 1.39, 1.4, 1.63, and 1.05 for CT-26, DLD-1, HT-29, and CRL-1831 cells, respectively. Also, C-PC + RT treated mice exhibited 35.2% lower mean tumors’ volume and about 6 days more survival time in comparison with the RT group (P < 0.05). In addition, C-PC + RT group exhibited 54% lower Ki-67 index in comparison with the RT group. Therefore, C-PC can exhibit high radiosensitizing effects. However, the potential cardiovascular risks of C-PC as a COX-2 inhibitor should be evaluated with extensive preclinical testing before developing this agent for clinical trials.

## Introduction

Colorectal cancer is known as a prevalent malignancy in the human societies^1^. Despite advance achievements in the field of colorectal cancer treatment, it remains one of the deathliest cancers worldwide. Radiation therapy is a part of the therapeutic approaches for locally advanced, locally recurrent, and oligometastatic colorectal tumors. Also, it can be used for palliative purposes^2^. At the rectal cancer management, radiation therapy plays the adjuvant treatment’s role to enhance the chemotherapy efficacy^3–6^.

Radiotherapy employs high energy radiation beams to damage malignant cells^2^. However, radioresistant properties of colon tumors significantly decrease the therapeutic efficacy^7^. Radioresistance means adaption of tumor cells to the radiation therapy-induced changes and developing of resistance to the radiation beams by utilizing multiple genes, factors, and mechanisms^8,9^. It is one of the main causes of radiation therapy failure in cancer patients which can cause poor prognosis^10^. Therefore, radiosensitizing agents have gained lots of attention for conquering this problem.

Radiosensitizers can significantly enhance radiation therapy efficacy. Different types of radiosensitizers have been introduced including chemical radiosensitizers, nanomaterials, etc.^11,12^. Recently, metal nanoparticles like gold nanoparticles have received lots of attention for radiosensitizing purposes^13,14^. However, many researchers believe that nanomaterials are far away from clinical applications due to lack of knowledge about their behavior inside the human body and unknown side effects^15,16^. Therefore, many studies have focused on nature originated drugs due to fewer side effects, high biocompatibility^17^, and better patients’ compliance in comparison with chemical agents and nanomaterials^18^. Many anticancer drugs which are originated from natural products have exhibited significant effectiveness for cancer treatment. These components are derived from plants, marine organisms, and microorganisms^19^.

C-phycocyanin (C-PC) is a biocompatible water-soluble biliprotein which exist in *Spirulina platensis*^20^. C-PC has exhibited potent various biological activities such as anti-oxidant^21^, radical scavenging^22^, and anti-inflammatory properties. Also, many studies have reported the anti-proliferative and anti-metastatic role of this protein against different cancer cell lines^23–25^. C-PC main mechanism of action is to inhibit cyclooxygenase-2 (COX-2) pathway^26–28^. COX-1 and COX-2 are the two recognized isoforms of COX. COX-1 is involved in controlling the normal tissues’ hemostasis. However, COX-2 expression has exhibited high correlation with tumor promoters, oncogenes, and carcinogens^29–31^. Many cancer types exhibited up-regulated expression of COX-2. In addition, COX-2 expression is associated with tumor grade, bad prognosis, and tumor invasion and metastasis^32–34^. Recently, many studies have identified the determinative role of COX-2 in the cancer cells radioresistant property. According to these studies, overexpression of COX-2 has a direct relation with cancer cells’ resistance to radiation beams. Also, inhibition of COX-2 caused significant enhancement at the radiation therapy efficacy and decreased the cancer cells’ radioresistance^35,36^.

Anti-tumor effect of C-PC at different cancers have been reported by different studies. Selective COX-2 inhibitory property of C-PC has been identified as one of the main reasons for its anti-tumoral effects. As COX-2 pathways are deeply involved in the radioresistance of cancer cells and this cancer cells’ property is one of the main reasons of treatment failure, we hypothesis that C-PC can act as a natural radiosensitizer for enhancement of radiation therapy efficacy. According to the best of our knowledge, this is the first time to use C-PC as the adjuvant treatment for enhancement of radiation therapy efficacy.

## Methods and Materials

### Cell culture and preparation

The CT-26 (murine colon cancer), DLD-1 (human colon cancer), HT-29 (human colon cancer), and CRL-1831 (normal human colonic cells) cell lines were purchased from the Pasteur Institute of Tehran, Iran. CT-26, DLD-1, and HT-29 cells were maintained in RPMI 1640 (Sigma, USA) supplemented with 10% fetal bovine serum (Sigma, USA) and penicillin-streptomycin (100 units/mL and 10 μg/mL, respectively) in the standard cell culture condition at 37 °C with 5% CO_2_. The CRL-1831 were cultured in DMEM-F12. When the cells’ confluence at the bottom of culture flask reached 80%, they were detached by 0.25% Trypsin and 0.001% EDTA solution (Sigma, USA) and then counted by Neubauer method and passaged to reach to the needed number of cells.

### Cell viability assay

The cells were seeded at 96-well plates at a density of 10^5^ cells per well and incubated for 24 hours. At first, the effect of different concentrations of C-PC on CT-26, DLD-1, HT-29, and CRL-1831 cell lines was investigated. Therefore, the cells were incubated with 0, 50, 100, and 200 µg/mL of C-PC for 24 h. Then, the C-PC was washed out by PBS and cells were incubated for another 24 h. After 24 h, the viability of the cells was evaluated using MTT kit according to the manufacturer’s instructions. The viability percent was expressed by relative value to the untreated cells (0 µg/mL). For each concentration at least 6 wells were used and the experiment was repeated three times. At the next step, the radiosensitizing effect of C-PC was evaluated at different radiation therapy dosages (2, 4, and 6 Gy). The cells were incubated with different concentrations of C-PC (0, 50, 100, and 200 µg/mL) for 24 h and then the wells were washed three times with PBS. After removing C-PC, the cells were irradiated by a Compact linear accelerator (Primus, Siemens Ltd, Germany). Source-to-surface distance (SSD) of 100 cm and field size of 25 × 25 cm^2^ were set. The plates were irradiated with 2,4, and 6 Gy with a dose rate of 200 MU/min. After 24 h, cellular viability was evaluated using the MTT assay kit. The viability percent was expressed by relative value to the cells which were not treated with C-PC or irradiation. To investigate the type of interaction between C-PC treatment and radiation therapy, the combination index (CI) was calculated based on the Chou and Talalay formula^37^ by using the obtained data from the MTT assay according to previous studies^38^.

### Clonogenic cell survival assay

The clonogenic cell survival assay was done based on previous studies^39^. Briefly, 10^5^ cells were seeded in 35 mm^2^ dishes and incubated for 24 h. Then, the cells were treated with C-PC (200 µg/mL) and incubated for 24 h. Subsequently, the cells were washed 3 times with PBS and irradiated with different doses of X-rays (0, 2, 4, 6, and 8 Gy). Immediately after irradiation, the cells were detached by trypsin and suspended in PBS to have a single cell suspension. The cells were counted by Neubauer method and the appropriate number of cells were reseeded in 100 mm^2^ Petri plates for 15 days. For each sample, at least three plates were prepared. At last, the cancer cells colonies were fixed by methanol and the fixed colonies were stained by crystal violet (0.5%). These colonies were counted by a loupe microscope (Olympus, Tokyo, Japan)^39,40^. The survival curves were drawn to obtain the dose enhancement factor (DEF)^41^. These investigations were done for all the used cell lines in the MTT assay including CT-26, DLD-1, HT-29, and CRL-1831. For more details please refer to our previous publication^42^.

### Animal husbandry and handling

70 Female BALB/c mice (age: 6–8 weeks, weight: 23 ± 2 g) were purchased from the Pasteur Institute of Tehran, Iran. The mice were maintained at 24 ± 2 °C temperature, 50 ± 10% relative humidity, and 12 h light/12 h dark cycle condition with complete access to standard mouse chow and water. The mice were acclimated for 10 days before the start of the study. If any signs of pain, wounds, massive tumor necrosis, hemorrhage, or diffuse metastasis were observed during any steps of the study, the mice were sacrificed by an overdose of ketamine/xylazine solution.

### Cancer cells implantation and tumor-bearing mice radiation therapy

**Figure 1:**
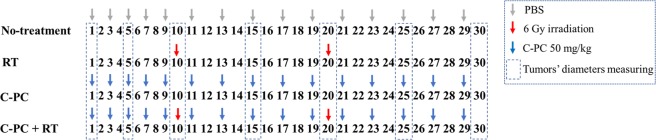
Schematic illustration of the therapeutic approaches for each group. The first day is when the tumors’ volume reached 50–70 mm^3^. Then, the tumors diameters were measured every 5 days for 30 days.

32 mice were involved in this experiment. The left flank of the mice was shaved and sterilized by 70% alcohol. Each mouse was injected subcutaneously with 1 × 10^6^ CT-26 cells suspended in 50 µL of DMEM-F12 (Sigma, USA) into its left flank. To determine tumors’ growth progression, the greatest longitudinal diameter (length) and the greatest transverse diameter (width) of the tumors were determined every 5 days. Then, the tumor’s volume was calculated by the tumor volume Eq. (1). When the tumors volume reached 50–70 mm³, the mice were randomly divided to 4 groups (n = 8) including PBS (no-treatment), C-PC, Radiation therapy (RT), and C-PC + RT. The mice at the 1^st^ group were injected with PBS. The 2^nd^ group was intraperitoneally (i.p) injected with C-PC 50 mg/kg once every other day during 30 days. The 3^rd^ group was irradiated with 6 Gy at the 10^th^ and 20^th^ days of the experiment. The C-PC + RT group was injected with C-PC 50 mg/kg once every other day during 30 days and, they were 6 Gy irradiated at the 10^th^ and 20^th^ days of the experiment. Before radiation therapy, the mice have intraperitoneally injected with a Ketamine-Xylazine (KX) solution (Ketamine: 190 mg/kg, Xylazine: 4 mg/kg) to immobilized them during irradiation. All the therapeutic approaches for each group are illustrated in Fig. 1. At the next step for survival analysis, all the groups were kept under observation for the next 60 days. The animals’ death was recorded every day. Standardized human endpoint used to euthanize animals was the failure to eat and drink for over 3 days and without any limb movement.

1 $${\rm{Tumor}}\,{\rm{volume}}=({\rm{Tumor}}\,{\rm{length}})\times {({\rm{Tumor}}{\rm{width}})}^{2}\times 0.52$$

### Flow cytometry

The CT-26 cells were incubated with different concentrations (50, 100, and 200 µg/mL) of C-PC (Sigma, USA) for 48 h. The C-PC was purchased from Sigma. Also, a group of cell-seeded wells were just irradiated with 6 Gy radiation (without C-PC treatment) and incubated for 24 h. Then, the cells were detached by trypsin and washed 3 times with PBS. Subsequently, they were fixed with paraformaldehyde (1%) for 10 min, followed by the addition of Triton X‐100 (0.3%) for another 10 min. Then, the cells were washed 3 times. The pellet of cells was suspended in PBS and stained with anti-COX-2 fluorescein isothiocyanate (FITC)‐conjugated monoclonal antibody (Cayman Chemical, USA). The cells were incubated with the antibody for 15 min in the dark. The unattached antibodies were discarded by centrifuging and PBS washing. Also, three samples of control CT-26 cells which were not incubated with anti-COX-2 antibody (unstained cells) were analyzed by flow cytometer (BD FACS Calibur, USA) for identifying the FL1-H+ and FL1-H – regions. The stained samples of control, 50, 100, 200 µg/mL treated cells were diluted in PBS and analyzed by flow cytometer. At least 5000 cells were analyzed from each sample. The results were analyzed using FlowJo (FlowJo, LLC, USA). Each group contained at least three samples and the experiment was repeated three times. The FL1-H- region was introduced to contain more than 99% of the unstained cells. Therefore, the FL1-H- will exhibit the COX-2 negative cells and the cells which express COX-2 will place at the FL1-H+ region.

### Western blot analysis

Anti-COX-2 antibody (ab179800, Abcam, USA) and anti-beta actin antibody (ab8227, Abcam, USA) were utilized for this study^43^. For *in vitro* evaluations of C-PC treatment effect on the COX-2 expression, CT-26 cells were incubated with different concentrations of C-PC (0, 50, 100, 200, and 300 μg/mL) in 6-well plates for 24 h. Subsequently, the cells were trypsinized and harvested after PBS washing. The protein concentrations of the samples were detected using a BCA protein assay kit (Abcam, USA). For *in vivo* evaluations, 10 female BALB/c mice were purchased and injected with CT-26 cells. When the tumors reached 50–70 mm^3^, the tumor-bearing mice were randomly divided into two groups (n = 5) including control and C-PC. The control group was injected with PBS. The C-PC group was i.p injected with C-PC (50 mg/kg) one every other day. The mice were sacrificed after 10 days and the tumors were harvested. Subsequently, the tumors were homogenized in RIPA buffer containing a proteinase inhibitor cocktail (Abcam, USA), sonicated, and incubated at 4 °C for 20 minutes on a rocking platform. Cell debris was removed by centrifugation and protein content was determined by Bradford assay. Proteins (40–80 µg) were separated on 10% SDS-PAGE gels and transferred onto nitrocellulose membranes. 4% milk protein in PBS/0.1% Tween-20 was employed for blocking of the membranes. The primary antibody was added to the same buffer and incubated overnight at 4 °C. Then, the anti-rabbit HRP-conjugated secondary antibody (ab6721, Abcam, USA) was added and incubated for one hour at the room temperature. Proteins were visualized on autoradiographic film using ECL reagent (Pierce). The MCF-7 cells which were cultured at 2-D culture were used as the negative control. Previous studies have used the lysed MCF-7 cells as a negative control for COX-2 expression analysis^44^.

### Immunohistochemistry

12 BALB/c mice were purchased and injected with CT-26 cells. When the tumors reached 50–70 mm^3^, the mice were randomly divided into 4 groups (n = 3) including PBS (no-treatment), C-PC, Radiation therapy (RT), and C-PC + RT. The treatment approaches were the same as section 2.6. The mice were sacrificed after 11 days and the tumors were harvested. Immunohistochemistry (IHC) was done according to previous studies^45^. Briefly, the tumors were fixed with 10% formalin and then, processed by employing an automatic tissue processor (Sakura, Japan). Then, the paraffin-embedded specimens were processed according to previous studies^46^ to be stained with anti-Ki-67 antibody (ab21700, Abcam, USA). Immuno-positive cells were quantified at random microscopic fields at ×400 magnification by an expert pathologist. A digital light microscope (Olympus, Tokyo, Japan) was used to capture the photographs.

### Quantitative real-time RT-polymerase chain reaction (qRT-PCR)

qRT-PCR was done as previous studies have described^47^. Briefly, CT-26 cells were incubated with different concentrations of C-PC (0, 50, 100, 200, and 300 μg/mL) in 6-well plates for 24 h. Subsequently, the cells were washed with PBS and harvested for total RNA extraction using the Trizol reagent following the manufacturer’s instructions. Primescript™ RT reagent kit was employed for reverse-transcribing RNA into cDNA. Rotor-Gene 3000 real-time PCR apparatus was used in this study. Also, the SYBR Green fluorescent dye method was utilized. COX-2 primer sequence (Invitrogen CO): 5- TCGATGTCATGGAACTGTA -3 (sense) and 5- TTCCAGTATTGAGGAGAAC -3 (anti-sense). beta-actin, its primer sequence was 5-GTTGCGTTACACCCTTTCTTG-3 (sense), 5-TGCTGTCACCTTCACCGTTC-3 (antisense). The relative expressions of COX-2 was assessed by utilizing Beta-actin as an internal control. The PCR conditions were as follows: a pre-denaturing at 95 °C for 2 min, followed by 45 cycles of denaturation at 95 °C for 10 s, annealing/extension at 60 °C for 20 s. The 2-ΔΔCT method was employed to calculate the relative abundance of the target gene expression. For each cDNA, the target gene mRNA level was normalized to beta-actin mRNA level. The experiments were performed in triplicate.

### Analysis of PGE2 synthesis

As previous studies have described^48^, CT-26 cells were seeded at 12-well plates for 12 h. Then, different concentrations of C-PC (0, 50, 100, 200, and 300 μg/mL) were added to culture media and incubated for 24 h. Subsequently, arachidonic acid was added to each well and after 1 h, the culture media were collected and cell derbies were removed by centrifuging. Prostaglandin E2 (PGE2) level in the cell-free culture medium was measured by employing PGE2 ELISA kits (Cayman Chemical Company, USA) according to the manufacturer’s instructions.

### Histopathology and blood biochemical assays

16 female BALB/c mice were randomly divided into 2 groups (n = 8) including PBS and C-PC groups. The mice at the 1^st^ group were injected with PBS. The 2^nd^ group was i.p injected with C-PC (50 mg/kg) once every other day during 30 days. The mice were closely monitored for the mortality, appearance, behavioral pattern changes such as weakness, aggressiveness, food or water refusal, and pain or any signs of illness within these 30 days. Also, the animals were weighed every 10 days to monitor their body weight. At the 30^th^ day, the mice were sacrificed and their blood was collected and the discarded serum was used for biochemistry evaluations. Blood urea nitrogen (BUN), creatinine (Cr), alanine aminotransferase (ALT), and aspartate aminotransferase (AST) were measured for biochemical assays. Moreover, the harvested organs were fixed, processed, sectioned, and stained by Hematoxylin & Eosin (H&E) according to previous studies^49,50^. Then, two pathologists reviewed the blindly-labeled slides. A digital light microscope (Olympus, Japan) was used to capture the slides’ histopathological photographs.

### Statistics and mathematical analyzes

The statistical analyses were performed by employing JMP 11.0 software (SAS Institute, Japan) and using one-way analysis of variance (ANOVA) with Tukey’s post-hoc test. To investigate the type of interaction between C-PC treatment and radiation therapy, the combination index (CI) was calculated based on the formula of Chou and Talalay^37^ and for this purpose, the data which were obtained from MTT assay were used according to previous studies^38^. The *in vitro* experiments were repeated at least three times and for each group in the *in vivo* experiments, at least 5 mice were included. The results were displayed as the mean ± standard deviation (SD). The difference was considered statistically significant if *P* < 0.05. (*P < 0.05, ns: not significant).

### Ethics statement

All experiments were done according to the Guidelines for the Care and Use of Laboratory Animals of Arak University of Medical Sciences, which refer to American Association for Laboratory Animals Science and the guidelines laid down by the NIH (NIH Guide for the Care and Use of Laboratory Animals) in the USA. All experimental protocols were approved by the ethics committee of Arak University of Medical Sciences.

## Results

### Radiosensitizing effect of C-PC treatment on the normal and cancerous cell lines *in vitro*

**Figure 2:**
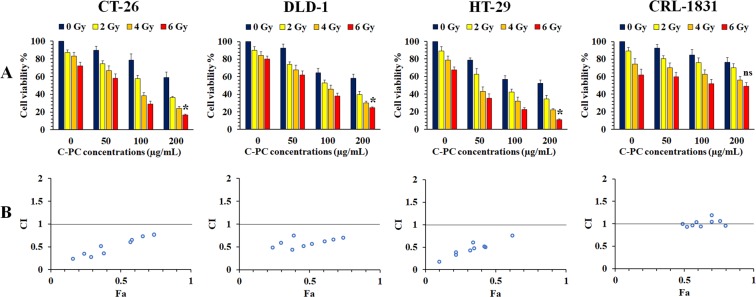
Investigation of the C-PC and radiation beams synergistic effects on the cancer and normal cells viability at different concentrations of C-PC (50, 100, and 200 µg/mL) and different radiation therapy dosages (2,4, and 6 Gy) by MTT assay and CompuSyn software. (**A**) Three different colon cancer cell lines including CT-26, DLD-1, HT-29 and CRL-1831 as the normal colonic cell line were used. (The first columns from the left side of the MTT assay charts exhibit the cell viability of the wells which were incubated with 0 µg/mL C-PC as the control (no-treatment). Also, the cell viability of the wells which were mono-treated with 2, 4, and 6 Gy radiation was illustrated in the 2^nd^, 3^rd^, and 4^th^ column from the left side of the MTT assay charts to exhibit the effect of radiation therapy mono-treatment on the cancer cells viability (*P < 0.05, ns: not significant). (**B**) To investigate, the synergistic effect of radiation therapy and C-PC co-treatment. The combination index (CI) was calculated by analyzing the MTT assay data for different cell lines using CompuSyn software. CI < 1, CI = 1, and CI > 1 stand for synergistic, additive, and antagonistic effects, respectively. Also, Fa refers to inhibitory rate.

To investigate the radiosensitizing effect of C-PC treatment, three colon cancer cell lines including CT-26, DLD-1, and HT-29 were selected. Also, CRL-1831 was used as the normal colonic cell line. A 96-well plate which was seeded by these cell lines was prepared and the cells were incubated with 0, 50, 100, and 200 µg/mL concentrations of C-PC for 24 h to exhibit the C-PC effect on the normal and cancerous colon cells. In this plate, the wells which were incubated with 0 µg/mL C-PC (the first column from the left side of the MTT assay charts) were used as the control. The other plates were irradiated with 2, 4, and 6 Gy X-ray radiation after 24 h incubation with different concentrations (0, 50, 100, and 200 µg/mL) of C-PC. The wells which were incubated with 0 µg/mL C-PC (the 2^nd^ column from the left side of the MTT assay charts) can exhibit the effect of RT mon-treatment on the cells’ viability. This experiment was repeated three times. As Fig. 2A illustrates, treatment with 200 µg/mL C-PC caused significant enhancement at the radiation therapy efficacy of all the colon cancer cell lines’ according to MTT assay. The lowest cancer cells’ viability was observed at the wells which were treated with 200 µg/mL C-PC + RT. Therefore, C-PC treatment can significantly sensitize the colon cancer cells to the radiation beams, especially at 200 µg/mL concentration. However, normal colonic cells treatment with C-PC didn’t cause significant (P > 0.05) impact on the radiation beams effect on these cells.

Also, the MTT assay obtained data were used to investigate the synergistic interactions between C-PC treatment and radiation therapy. These data were used to determine whether co-treatment with C-PC and radiation therapy were interacting in a synergistic or additive manner based on the Chou and Talalay formula^37,38,51^. As Fig. 2B illustrated, the calculated combination index (CI) indicated a synergistic interaction between C-PC treatment and radiation therapy for colon cancer cell lines. But this interaction wasn’t synergistic for CRL-1831 cells as the normal colonic cell line.

**Figure 3:**
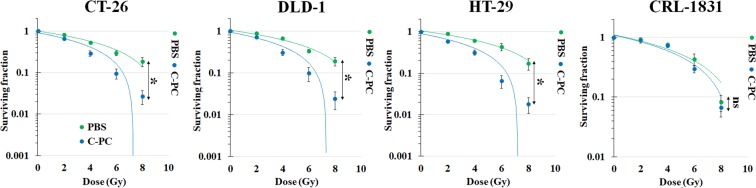
Investigation of the radiosensitizing effect of C-PC pre-treatment by clonogenic cell viability assay for three different colon cancer cell lines (CT-26, DLD-1, and HT-29) and a normal colonic cell line (CRL-1831). (*P < 0.05, ns: not significant).

To validate the MTT assay results, the radiosensitizing effect of C-PC was evaluated by clonogenic cells’ viability assay (Fig. 3) and the most effective concentration of C-PC treatment according to MTT assay (200 µg/mL) was selected for this experiment. The acquired DEF for treatment with 200 µg/mL C-PC was 1.39, 1.4, 1.63, and 1.05 for CT-26, DLD-1, HT-29, and CRL-1831 cells, respectively. Therefore, C-PC at this concentration can significantly sensitize the colon cancer cells to radiation beams and enhance radiation therapy efficacy. However, no radiosensitizing effect was observed after C-PC treatment for the normal colonic cells.

### Radiosensitizing effect of C-PC treatment on the CT-26 colon cancer cells *in vivo*

**Figure 4:**
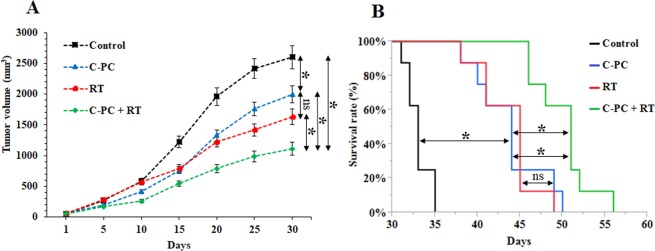
Evaluation of the radiosensitivity effect of C-PC treatment on of the CT-26 colon tumors *in vivo*. (**A**) The tumors’ growth progression and (**B**) tumor-bearing mice survival time at different groups (n = 8) including Control, C-PC, RT, and C-PC + RT. (*P < 0.05, ns: not significant).

The tumor growth progression and survival time were investigated at 4 groups (n = 8) of CT-26 tumor-bearing mice including PBS (no-treatment), C-PC, Radiation therapy (RT), and C-PC + RT which were underwent different therapeutic regimes (Fig. 1). The mice at the 1^st^ group were injected with PBS. The 2^nd^ group was treated with 50 mg/kg C-PC, i.p, once every other day during 30 days. This dosage of C-PC treatment was selected according to the previous studies which have used C-PC as the main treatment for inhibition of tumors’ growth^28^. The 3^rd^ group was irradiated with 6 Gy at the 10^th^ and 20^th^ days of the experiment. The C-PC + RT group was injected with C-PC 50 mg/kg once every other day during 30 days and, they were 6 Gy irradiated at the 10^th^ and 20^th^ days of the experiment (6 Gy, two times with 10 days interval). As Fig. 4A illustrates, the C-PC treatment could significantly inhibit the tumors’ growth in comparison with the no-treatment group. However, the slowest tumors’ growth progression was observed at the tumors of mice which were treated with the combination of C-PC and radiation therapy. Although radiation therapy could significantly inhibit the tumors’ growth, the combination of C-PC and radiation therapy treatment (50 mg/kg once every other day for 30 days plus two times radiation with 10 days interval) caused a significantly higher therapeutic effect. At the last day of measuring tumor’s diameters (30^th^ day), the mean tumors’ volume for the control group was 1960 ± 140 mm^3^. The C-PC treatment, RT, and C-PC + RT groups exhibited a 32.1%, 37.7%, and 59.6% decrease in the tumors volume in comparison to the control. These observations can demonstrate the radiosensitizing effect of C-PC treatment for enhancement of radiation therapy efficacy. In addition, the effect of each therapeutic approach was investigated on the tumor-bearing mice survival time (Fig. 4B, Table 1). The mean survival time for the control group was 33 ± 1.4 days. C-PC treatment caused about 10 days increase in the tumor-bearing mice survival time in comparison with control. However, the tumor-bearing mice at the C-PC + RT group exhibited 50.1 ± 3.3 days mean survival time which was significantly (*P < *0.05) more than all other groups.

**Table 1:** Evaluation of the tumor-bearing mice mean survival time in different groups.

Groups	Survival time (Days)
Control	33 ± 1.4
C-PC	43.7 ± 4.1
RT	43.6 ± 3.4
C-PC + RT	50.1 ± 3.3

Control: No-treatment group, RT: Radiation therapy, C-PC: C-phycocyanin. C-PC + RT: Combination of C-PC treatment and radiation therapy.

**Figure 5:**
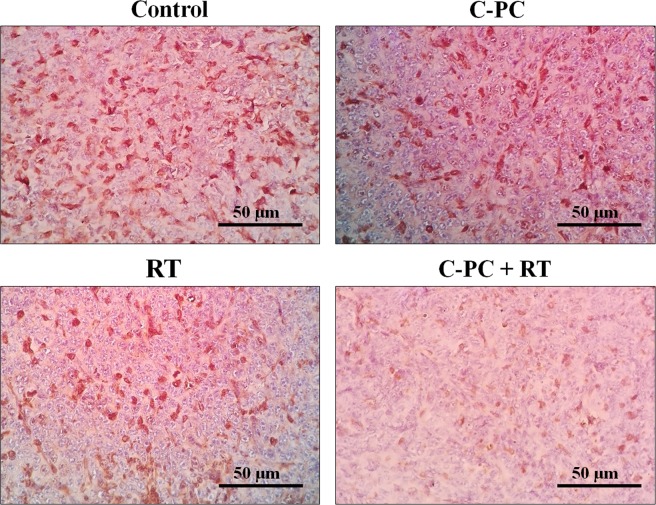
Light microscopy photographs of the immunostained sections of CT-26 tumors at different groups (Control, C-PC, RT, and C-PC + RT) on the 11^th^ day of treatment (n = 3).

Ki-67 is a marker which indicates the proliferating cells and the Ki-67 index can quantitatively estimate the cancer cells’ proliferation rate at the tumor. As Fig. 5 illustrated, the Ki-67 index was 56.4 ± 6.1% for the no-treatment group (n = 3), 31.6 ± 9.4% for the C-PC group (n = 3), 27.8 ± 7.3% for the RT group (n = 3), and 12.8 ± 5.1% for the C-PC + RT group (n = 3). Therefore, C-PC pre-treatment (C-PC + RT) could significantly (*P* < 0.05) decrease the Ki-67 index in comparison with no-treatment, C-PC, and RT groups. These results are inconsistent with the tumors’ growth progression and mean survival times at the different groups.

### Predominant mechanism in mediating radiosensitizing effect of C-PC

**Figure 6:**
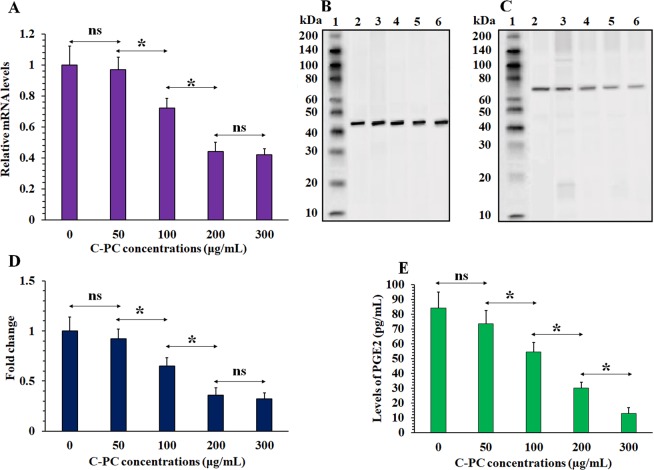
Evaluation of C-PC treatment effect on the COX-2 expression in the CT-26 cells. (**A**) The relative levels of COX-2 mRNA at the CT-26 cells after treatment with different concentrations of C-PC (0, 50, 100, 200, and 300 µg/mL) according to qRT-PCR analyses. (**B**) western blot analyses of beta-actin and (**C**) COX-2 proteins expression after treatment with different concentrations of C-PC (0, 50, 100, 200, and 300 µg/mL). The 1, 2, 3, 4, 5, and 6 lanes represent the protein marker, 0, 50, 100, 200, and 300 µg/mL samples, respectively. The figure displays the full-length blots with no cropping. (**D**) The normalized COX-2 protein levels at different treatment groups (0, 50, 100, 200, and 300 C-PC µg/mL). (**E**) The PGE2 level at the CT-26 cells’ culture media after 24 h incubation with different concentrations of C-PC (0, 50, 100, 200, and 300 µg/mL). (*P < 0.05, ns: not significant).

Previous studies have attributed most of the therapeutic properties of C-PC to two mechanisms including direct COX-2 inhibition and downregulating COX-2 expression^26,27,47^. However, determining the predominant axis between these two mechanisms in mediating the synergism between C-PC and radiation therapy is very important as direct COX-2 inhibition can cause major cardiovascular toxicity and many direct COX-2 inhibitors has been prevented from clinical utility^52,53^. For this purpose, the levels of COX-2 mRNA (Fig. 6A) and protein (Fig. 6B–D) were measured by qRT-PCR and western blot, respectively. COX-2 mRNA was highly expressed in the CT-26 cancer cells and C-PC treatment caused significant decrease in the COX-2 mRNA and protein levels. The most suppression of COX-2 mRNA and protein expression was observed at 200 and 300 µg/mL C-PC treated cells. However, no significant (P > 0.05) difference was observed between these two concentrations for inhibition of COX-2 expression. In addition, the levels of prostaglandin E2 (PGE2) as the main product of COX-2 enzyme activity was measured at the cell culture media. As illustrated in Fig. 6E, the PGE2 level for the control (0 µg/mL), 50, 100, 200, and 300 µg/mL C-PC treated cells culture media were 84.1 ± 10, 73.3 ± 9, 54.2 ± 4, 29.2 ± 4, and 12.8 ± 3 pg/mL, respectively. Therefore, 50, 100, 200, and 300 µg/mL C-PC treatment caused about 12, 35, 64, and 84% decrease at the PGE2 levels in comparison with control (0 µg/mL C-PC), respectively. On the other hand, 50, 100, 200, and 300 µg/mL C-PC treatment caused about 6, 28, 56, and 58% decrease in the mRNA levels of COX-2 in comparison with control (0 µg/mL) and also, about 8, 30, 64, and 68% decrease in the COX-2 protein levels. Therefore, It seems that in all of the C-PC concentrations the main determinative mechanism for decrease of PGE2 level is decrease of COX-2 expression. 200 and 300 µg/mL concentrations exhibited no significant difference at inhibition of COX-2 mRNA and protein expression. However 300 µg/mL concentration exhibited significantly higher decrease in the PGE2 level in comparison with 200 µg/mL which can be attributed to increase of direct COX-2 inhibition effect of C-PC at this concentration.

### Inhibition of COX-2 expression by C-PC treatment *in vivo*

**Figure 7:**
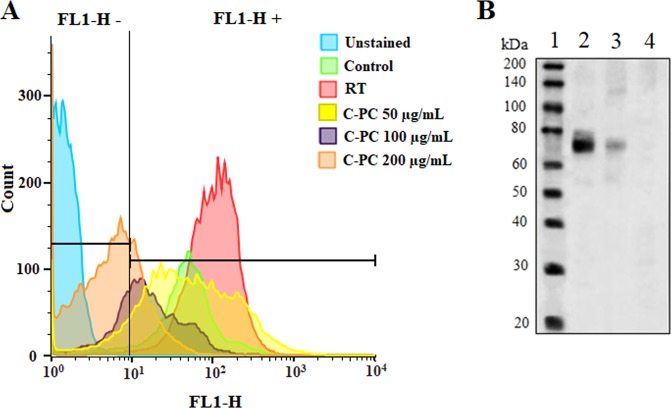
Investigation of C-PC effect on the cancer cells’ COX-2 expression *in vitro* and *in vivo*. (**A**) Flow cytometry analyses of COX-2 expression at the CT-26 cancer cells after incubation with different concentrations of C-PC (50, 100, and 200 µg/mL) and radiation therapy. The control cells were treated with PBS. The unstained cells were not incubated with anti-COX-2 antibody to identify the FL1-H+ and FL1-H- regions (FL1-H+: COX-2 positive region, FL1-H-: COX-2 negative region). Also, a group of wells was just irradiated (6 Gy) to identify the radiation therapy effect on COX-2 expression. (**B**) Western blot analysis of COX-2 expression in the tumors of C-PC treated (lane 3) tumor-bearing mice. Lane 2 exhibits the no-treated tumor-bearing mice’s tumors as positive control. The lanes 1 and 4 are presenting the ladder and the lysed MCF-7 cells as the negative control, respectively. The figure displays the full-length blots with no cropping.

The inhibitory effect of C-PC treatment on the CT-26 cells’ COX-2 expression was evaluated *in vitro* and *in vivo*. For *in vitro* evaluations, the CT-26 cells were incubated with different concentrations of C-PC (50, 100, and 200 µg/mL) for 48 h. Then, the COX-2 expression at the inner of the cancer cells was evaluated by flow cytometry (Fig. 7A). At first, the control cells which were not incubated with C-PC were analyzed. As illustrated in Table 2, COX-2 was expressed by more than 92% of the untreated CT-26 cells. However, this percentage decreased to 88.2 ± 7.6%, 71.6 ± 6.1%, and 25.2 ± 9.4% after treatment with 50, 100, and 200 µg/mL of C-PC, respectively. Also, the COX-2 expression at the 6 Gy irradiated CT-26 cells exhibited no significant (*P* > 0.05) difference in comparison with control. Taking together, it seems treating of the CT-26 colon cancer cells with C-PC can significantly cause inhibition of COX-2 expression. However, RT per se doesn’t significantly affect COX-2 expression.

**Table 2:** Evaluation of C-PC treatment effect on the COX-2 expression in the CT-26 cells by flow cytometry.

Groups	COX-2 negative %	COX-2 positive %
Control	7.2 ± 4.3%	92.8 ± 4.3%
RT	1.7 ± 1.1%	98.3 ± 1.1%
C-PC 50 µg/mL	11.8 ± 7.6%	88.2 ± 7.6%
C-PC 100 µg/mL	28.4 ± 6.1%	71.6 ± 6.1%
C-PC 200 µg/mL	74.8 ± 9.4%	25.2 ± 9.4%

Control: The cells which were not treated with C-PC (0 µg/mL), C-PC: C-phycocyanin., RT: radiation therapy.

At the next step, the inhibitory effect of C-PC treatment on the CT-26 colon tumors’ COX-2 expression was evaluated *in vivo*. The tumor-bearing mice were i.p injected with C-PC 50 mg/kg once every other day for 10 days. This dosage of C-PC treatment was selected according to the previous studies which have used C-PC as the main treatment for inhibition of tumors’ growth^28^. The western blot analyses exhibited high expression of COX-2 protein at the CT-26 tumor which was significantly inhibited by C-PC treatment (Fig. 7B). Therefore, C-PC treatment can significantly decrease COX-2 expression at the CT-26 colon tumors.

### Safety of high dose C-PC treatment

**Figure 8:**
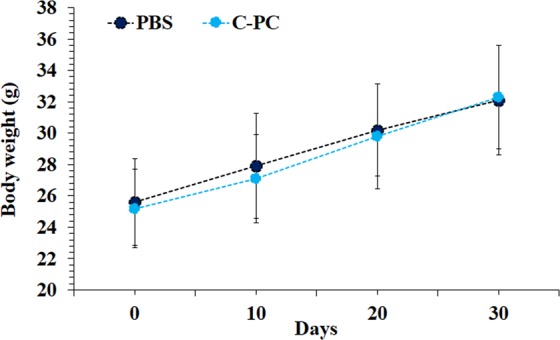
Monitoring of C-PC multiple high dose injections effect on the non-tumor bearing BALB/c mice weight during 30 days treatment period.

**Figure 9:**
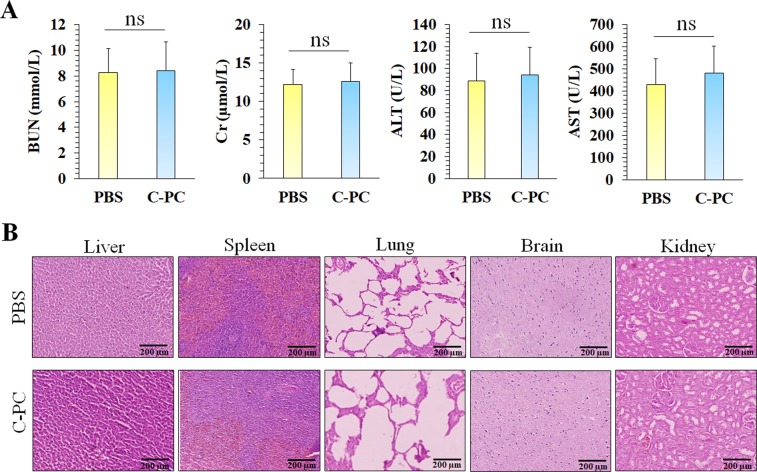
Histopathological exams and blood biochemical analyses of the mice treated with PBS and C-PC (n = 8) for the evolution of C-PC treatment safety. (**A**) Blood urea nitrogen (BUN), creatinine (Cr), alanine aminotransferase (ALT), and aspartate aminotransferase (AST) were measured as blood biochemical analyzes. (**B**) Photographs of histopathological sections from liver, spleen, lungs, brain, and kidney of the mice at PBS and C-PC treated groups (All scale bars are 200 µm). (*P < 0.05, ns: not significant).

Although many studies have demonstrated the safety of C-PC^54–56^, applying a high dose of C-PC (50 mg/kg) can cause some concerns about its probable side effects in high doses treatment. Therefore, BALB/c mice were injected with C-PC (50 mg/kg, i.p) once every other day during 30 days and the animals were completely monitored during this time period. No sign of changes at the appearance and behavioral pattern of the mice were observed. In addition, no significant difference in the bodyweight of the C-PC injected and control animals were detected (Fig. 8). At the 30^th^ day, the mice were sacrificed and their plasma was collected for biochemical (Fig. 9A) analyzes. Also, their organs including (lungs, liver, kidney, brain, and spleen) were harvested for histopathological investigations (Fig. 9B). No sign of organ damage or toxicity was observed at histopathological evaluations of the identified organs in the C-PC injected animals.

## Discussion

Radiation therapy employs high energy radiation beams to damage cancer cells through direct or indirect effects. Radiation beams’ direct interactions with DNA can cause single and double-strand breaks. Also, radiation therapy generates reactive oxygen species (ROS) from the radiolysis of water which can destroy biomolecules known as indirect effects. Therefore, these direct and indirect effects can change gene expression pattern and cellular signaling pathways for apoptosis induction or activation of pro-survival mechanisms to determine the cancer cells’ fate^57–59^.

Radioresistance means cellular resistance to the activation of apoptosis signaling pathways after irradiation^60,61^. One of the most well-known and determinative agents for radioresistance properties of cancer cells is COX-2. Induction or overexpression of COX-2 causes inhibition of cancer cells’ apoptosis^62^, resistance to treatments^63^, and proliferation^64^. COX-2 is the inducible member of the cyclooxygenase’s enzymes family. This enzyme is located at the luminal side of the endoplasmic reticulum and nuclear membrane. It plays a key role in prostaglandin and other eicosanoids biosynthesis from arachidonic acid^65^. Different growth factors and cytokines including IL-1β, IL-6, or TNF-α are involved in the regulation of COX-2 gene expression^66^. Besides, the COX-2 gene promoter contains an NF-κB response element as well as mentioned cytokines-dependent (i.e., IL-6) response elements^65^. Also, ROS production of radiation therapy per se can up-regulate COX-2 gene expression through activation of NF-κB^67,68^. According to previous studies, COX-2 overexpression cause production of prostaglandins (PG) which is known to modulate cell proliferation and cell death in many types of cancer including the colon. PGs act through different membrane receptors called EP receptors leading to activation of different pathways including β-catenin, a pathway which activates cancer cells’ proliferation^69^. Also, PGs can inhibit apoptosis in the cancer cells through up-regulation of the anti-apoptotic members of the Bcl_2_ family. On the other hand, COX-2 can stimulate the expression of ERK and PI-3K/Akt through stimulation of the EGFR pathway which leads to up-regulation of Bcl-2 proteins^70^. Also, up-regulation of PI-3K deactivates the Bcl-2 agonist of cell death (BAD) gene expression which is a pro-apoptosis gene^71^. Taken together, radiation therapy causes different destructions in the cancer cells like DNA damage which activate different pathways including DNA damage pathways (i.e., p53) to induce apoptosis. In contrast, COX-2 can inhibit apoptosis process through the production of PGs in the cancer cells which inhibit apoptosis and activate cancer cells’ proliferation through different pathways as mentioned above. Therefore, COX-2 induction or overexpression can cause significant radioresistance at the cancer cells^72^.

C-PC is a natural product and many studies have attributed its therapeutic effects to inhibition of COX-2 expression and activity^26,27,73–76^. On the other hand, COX-2 plays undeniable role in cancer radioresistance. Therefore, our team focused on the effects of C-PC on the COX-2 pathway and determining the predominant mechanisms in mediating the synergism between C-PC and radiation therapy^26,27,47^. The mechanism of direct COX-2 activity inhibition by C-PC appears to be the same as the selective COX-2 inhibitor drugs (e.g. celecoxib). This inhibition is caused via formation of a complex at the active site of the COX-2 enzyme^77,78^. COX-2 active site can accommodate bigger structures in comparison with COX-1 due to its larger size^79^. Therefore, bigger size of C-PC (~37.5 kDa) in comparison with non-steroidal anti-inflammatory drugs (NSAIDs) and also, its three-dimensional structure can ease its binding to the COX-2 active site^80^. In accordance to this fact, many studies have reported more potency of C-PC (IC50: 180 nM) for inhibition of COX-2 activity in comparison with celecoxib (IC50: 255 nM) and rofecoxib (IC50: 401 nM)^27,81,82^. As mentioned above, COX-2 gene up-regulation depends on different agents, including inflammatory cytokines (i.e., TNF-α, IFN-γ, IL-1, and IL-6) and oncogenes (such as Wnt/β-catenin)^36,83,84^. Many studies have demonstrated the inhibitory effect of C-PC on these factors’ activity including IL-6, IL-1, IFN-γ, TNF-α, and Wnt/β-catenin. In addition, C-PC can reduce NF-κB signaling activity which has a well-known binding site for COX-2 gene promotor^24,85,86^. Therefore, C-PC not only directly inhibits COX-2 activity but also, decrease COX-2 protein expression.

In this study, C-PC was used for enhancement of colon cancer radiation therapy through COX-2 expression inhibition as a natural radiosensitizer. The used C-PC dosage was selected according to the previous studies which have used C-PC as the main treatment for inhibition of tumors’ growth *in vivo*. Lia *et al*. investigated 12.5, 25, and 50 mg/kg C-PC for inhibition of pancreatic cancer tumor’s growth *in vivo* and observed the best therapeutic efficacy at 50 mg/kg^28^. In addition, no toxicity was reported in these dosages by the authors. In this study, histopathological and blood biochemical evaluations did not exhibit any damage to liver, lungs, spleen, brain, and kidney due to C-PC treatment (Fig. 9). According to other studies, administration of C-PC at the high doses from 250 to 500 mg/kg body weight (w/w) does not induce obvious symptoms nor mortality in animals^55^. Also, clinical safety of high dose phycocyanin (~1000 mg/day) was demonstrated by Jensen *et al*.^54^. Taken together, these observations suggest the relevant potential of high dosage C-PC for further cancer-related clinical trials. However, the potential cardiovascular risks of C-PC treatment as a COX-2 inhibitor should be evaluated with extensive preclinical testing before developing this agent for clinical trials. This issue is important because COX-2 down regulation in normal endothelial cells is deeply related to increasing risk of cardiovascular diseases which was observed with other COX-2 inhibitors (e.g. Celecoxib). Selective suppression of vasodilator and platelet inhibitory prostaglandins without blocking the vasoconstrictive and platelet-activating prostaglandins by COX-2 inhibitors can enhance the risk of hypertension, atherosclerosis, or even thrombosis^87–90^. Therefore, comprehensive preclinical experiments for investigating the C-PC safety in relation to cardiovascular toxicity is necessary in further studies.

## Conclusions

C-PC is an anti-cancer agent with the natural origin which has a long history of application as a food supplement. Many studies have reported anti-cancer properties for C-PC and demonstrated that C-PC exerts its effect through different mechanisms including COX-2 inhibition. According to this study, C-PC can significantly enhance the radiation therapy efficacy at colon cancer cells *in vitro* and *in vivo*. Therefore, C-PC not only can inhibit cancer cells proliferation but also sensitizes them to radiation beams. Its natural origin, long history of food supplement application, significant anti-tumor effects, and radiosensitizing properties can facilitate its evaluation at clinical trials. However, the potential cardiovascular risks of C-PC treatment as a COX-2 inhibitor should be evaluated with extensive preclinical testing before developing this agent for clinical trials.
